# Ni Nanoparticles Stabilized by Hyperbranched Polymer: Does the Architecture of the Polymer Affect the Nanoparticle Characteristics and Their Performance in Catalysis?

**DOI:** 10.3390/ijms232213874

**Published:** 2022-11-10

**Authors:** Svetlana A. Sorokina, Nina V. Kuchkina, Mariam G. Ezernitskaya, Alexey V. Bykov, Alexander L. Vasiliev, Nikolay N. Efimov, Zinaida B. Shifrina

**Affiliations:** 1 A.N. Nesmeyanov Institute of Organoelement Compounds, Russian Academy of Sciences, 28 Vavilov Street, Moscow 119991, Russia; 2 Department of Biotechnology and Chemistry, Tver State Technical University, 22 A. Nikitina Street, Tver 170026, Russia; 3 Shubnikov Institute of Crystallography of Federal Scientific Research Centre, Crystallography and Photonics of Russian Academy of Sciences, Leninsky Prospect, 59, Moscow 119333, Russia; 4 Kurnakov Institute of General and Inorganic Chemistry, Russian Academy of Sciences, Leninsky Prospect, 31, Moscow 119334, Russia

**Keywords:** nickel nanoparticles, hyperbranched polymer, catalysis, Suzuki-Miyaura cross-coupling, magnetic properties

## Abstract

Heat-up and hot-injection methods were employed to synthesize Ni nanoparticles (NPs) with narrow size distribution in the presence of hyperbranched pyridylphenylene polymer (PPP) as a stabilizing agent. It was shown that depending on the synthetic method, Ni NPs were formed either in a cross-linked polymer network or stabilized by a soluble hyperbranched polymer. Ni NPs were characterized by a combination of transmission electron microscopy (TEM), scanning TEM, thermogravimetric analysis, powder X-ray diffraction, X-ray photoelectron spectroscopy, energy dispersive X-ray analysis, and magnetic measurements. The architecture of polymer support was found to significantly effect Ni NPs characteristics and behavior. The Ni NPs demonstrated a high catalytic activity in a model Suzuki–Miyaura cross-coupling reaction. No significant drop in activity was observed upon repeated use after magnetic separation in five consecutive catalytic cycles. We believe that hyperbranched PPP can serve as universal platform for the controllable synthesis of Ni NPs, acting as highly active and stable catalysts.

## 1. Introduction

The development of stabilized transition-metal nanoparticles (NPs) as catalysts is one of the important areas of organic synthesis. Such catalysts occupy a niche between homogeneous and heterogeneous catalysts and can be widely employed for the synthesis of valuable chemicals. A high surface-to-volume ratio characteristic of NPs provides the interaction of reacting molecules with many catalytic centers on the NP surface, allowing for effective catalysis [1,2,3,4,5].

The nature of the support has a strong effect on the NPs stabilization against aggregation and influences the NPs catalytic activity. Encapsulation of NPs by macromolecules of a hyperbranched architecture is a promising approach to composite nanoparticulate catalysts [3,6,7]. Due to their spatially highly branched architecture, with plentiful functional (coordinating) groups and internal cavities, such macromolecules act as stabilizing and capping ligands, providing a confined space that limits NP growth and allows the avoidance of NP aggregation. At the same time, this architecture provides accessibility of the entrapped catalytic NPs for the reactants, leading to enhanced catalyst performance [6,8,9,10,11,12,13,14,15,16]. Moreover, a sterically demanding hyperbranched structure provides an unpassivated surface of NPs, thus contributing to the enhancement of their catalytic activity.

The recovery and reusability of the catalyst represents a key issue for the sustainable development of a catalytic process. Catalysts that are active but difficult to recover for repeated uses are normally not chosen in industry. Usually, magnetic NPs as catalyst support offer robust separation of the catalysts from the reaction mixture, with an external rare-earth magnet without a filtration step [17].

The high-temperature decomposition of organometallic precursors in the presence of stabilizing and capping molecules is one of the most effective synthetic procedures allowing a synthesis of NPs with a narrow NP size distribution [18]. Surfactants, polymers, porous SiO_2_, etc., can be used as protecting and stabilizing agents for NPs. The use of thermally stable media is required for this approach, since the metal precursor decomposition temperature is usually above 200 °C. Previously, we synthesized magnetic iron oxide NPs via the decomposition of Fe nitrate in mesoporous silica in the presence of ethylene glycol followed by coating the silica surface with hyperbranched functional macromolecules and the formation of Pd catalytic species in the functional environment attached to the silica surface. Such a design of the catalytic composite provided an excellent catalytic performance in a model Suzuki–Miyaura reaction with efficient reusability due to magnetic separation [19,20,21]. In another study, the thermal decomposition of Ru(acac)_3_ in the reaction solution of a pre-synthesized hyperbranched functional polymer led to the formation of well-defined Ru NPs catalytically active in hydrogenation of levulinic acid into gamma-valerolactone [22].

While Pd NPs show the best performance in cross-coupling reactions [2,23,24,25,26], there is a serious concern about the future availability of Pd due to high demand and limited resources, as well as high cost and environmental dissipation. This encourages efforts directed towards a Pd replacement by more abundant and inexpensive metals, such as Ni, Cu, and Co [27,28,29,30,31,32]. In the case of Ni NPs, both catalytic and magnetic features are combined in the same NPs, which offers a more economically and environmentally favorable approach for the development of catalytic systems.

In this paper we focused on syntheses of Ni NPs by two synthetic protocols—thermal decomposition of Ni(acac)_2_ by heat-up [18,33,34,35,36] or hot-injection [37,38] techniques—in the presence of hyperbranched pyridylphenylene polymer (PPP) as stabilizing media. We studied the differences in characteristics of NPs prepared by these methods and the behavior of the catalysts developed in the model Suzuki–Miyaura cross-coupling reaction. It is noteworthy that the PPP synthesized by us earlier [39] was chosen due to its high thermal stability and ability to stabilize metal NPs [21]. It was found that PPP provided two types of the polymer support depending on the protocol of NP preparation, which is reflected in different characteristics and properties of Ni NPs. The Ni NPs demonstrated an excellent activity and selectivity in the model Suzuki–Miyaura reaction. The magnetic properties of Ni NPs have an added advantage for the catalysts, allowing magnetic separation and repeated uses without noticeable loss of catalytic activity during five consecutive catalytic cycles. We believe that PPP can serve as universal matrix that provides control over the NP synthesis and the catalytic properties.

## 2. Results and Discussion

### 2.1. Ni NP Formation. Morphological and Structural Characteristics vs. a Preparation Method

**Figure 1:**
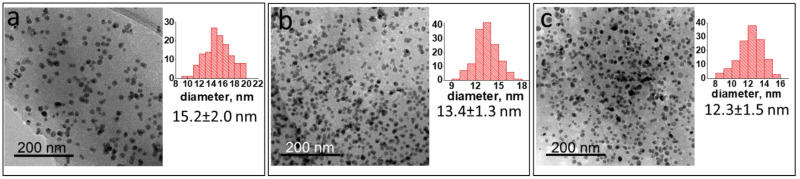
TEM images and size distributions of Ni NPs@PPP prepared by the heat-up approach at Ni(acac)_2_/PPP ratio = 1 mmol/0.148 g, for 30 min at 200 °C (**a**), 220 °C (**b**) and 280 °C (**c**).

**Figure 2:**
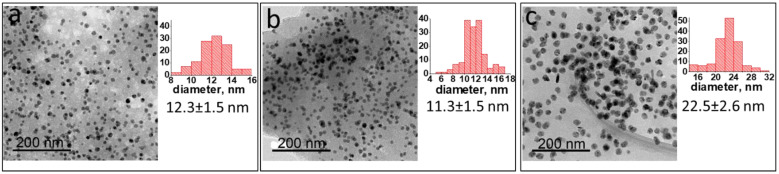
TEM images and size distributions of Ni NPs@PPP prepared by the heat-up approach at Ni(acac)_2_/PPP ratio = 0.5 mmol/0.148 g, for 30 min at 220 °C (**a**) and 280 °C (**b**) and Ni(acac)_2_/PPP = 0.5 mmol/0.0206 g for 30 min at 220 °C (**c**).

**Figure 3:**
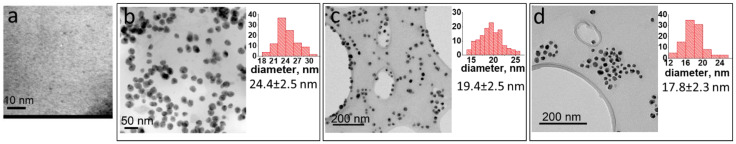
TEM images and size distributions of Ni NPs prepared by the injection approach at 220 °C (**a**), 240 °C (**b**), 260 °C (**c**) and 280 °C (**d**) within 30 min at Ni(acac)_2_/PPP = 0.5 mmol/0.148 g.

For further discussion, the two protocols for Ni NP preparation were denoted as “heat-up” and “injection”. The transmission electron microscopy (TEM) images of the NPs synthesized by both methods show spherical NPs surrounded by light grey polymer film (Figure 1, Figure 2 and Figure 3).

For Ni NPs synthesized by the heat-up approach (Figure 1), when the reaction temperature increases at the same precursor loadings and reaction time, the size of Ni NPs slightly decreases, indicating the effect of the reaction temperature on NP formation.

An increase in the PPP amount relative to the Ni precursor led to a minor decrease in the NP sizes for all temperature regimes at the same reaction time (compare Figure 2a,b with Figure 1b,c). The more pronounced effect of the polymer as stabilizing agent was gained from the samples synthesized at a significantly reduced amount of PPP (Figure 2c). When the PPP amount was reduced by seven times, much larger NPs (22.5 ± 2.6 nm) were generated within 30 min at 220 °C, revealing an important role of the polymer in NP stabilization.

An increase in the reaction time from 30 to 60 min had a little effect on the NP size, which indicates the process is mainly completed within 30 min (data not shown). It is important to note that the thermal decomposition of Ni(acac)_2_ in the presence of PPP was accompanied by the formation of a polymer gel in the temperature range of 160–200 °C. We suggest a cyclotrimerization of the residual ethynyl termini of PPP promoted by the Ni species is occurred during the procedure [40,41,42]. More discussion on this issue is provided in the section below.

The more noticeable effect of the temperature regime on the Ni NP formation was observed when the Ni compound was injected in the pre-heated polymer solution and was maintained at the desired temperature for the chosen time (injection approach). The TEM images in Figure 3 show the Ni NPs generated at different temperatures but for the same time and at the same Ni(acac)_2_: PPP ratio. Surprisingly, unlike the heat-up approach, no NPs were observed when the Ni precursor was injected at 200 °C and the reaction was stirred for 30 min (not shown), while at 220 °C only a few NPs of 1–2 nm were detected (Figure 3a). Upon the increase in the injection temperature to 240 °C, the NPs grow to 24.4 nm within 30 min (Figure 3b). The further temperature increase to 260 °C and 280 °C has led to a decrease in the NP size (for the same reaction time) (Figure 3c,d). It is worth noting that no polymer gel was observed for the injection protocol, allowing us to assume a different pathway for the formation of Ni NPs@PPP nanocomposite compared to the heat-up approach.

**Figure 4:**
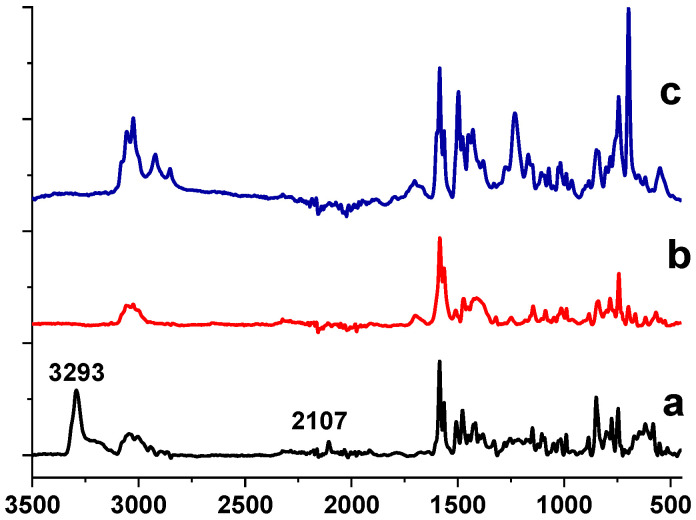
FTIR spectra of the dendrimer (**a**, black line), the composite of dendrimer with Ni(acac)_2_ prepared at 240 °C (**b**, red line) and PPP heated at 200 °C for 1 h (**c**, blue line).

To clarify such a possibility, a model experiment was carried out. Ni(acac)_2_ was heated in the solution of a model dendrimer (Figure S1) containing ethynyl end groups, which serves as a starting compound for the PPP synthesis. The structure of this dendrimer is close to that of the PPP monomer unit. We have observed the gelling process starting at 145 °C, which resulted in a completely crosslinked gel at 175 °C. In the FTIR spectrum of this composite sample, bands at 2100 cm^−1^ and 3300 cm^−1^ corresponding to vibrations of -C≡CH end groups of the dendrimer were absent (Figure 4, compare black and red lines), suggesting the formation of the crosslinked structure due to the cyclotrimerization of triple bonds catalyzed by Ni(acac)_2_ species [40,41,42]. Additionally, the characteristic C=O band at 1594 cm^−1^ normally observed for Ni(acac)_2_ and the broad band at 3408 cm^−1^, corresponding to crystalline water, were also absent [43] (Figure 4, red line), revealing no traces of acetylacetonate residues from Ni(acac)_2_ in the FTIR spectrum of the composite.

These data obtained for the model system allow us to propose the following pathway for Ni NPs@PPP. Ni(acac)_2_ promotes a cyclotrimerization process with the formation of the polymer network at the temperature below the thermal decomposition of Ni(acac)_2_ (160–180 °C). The continuous heating of the reaction mixture until a desired temperature leads to the thermal decomposition of Ni(acac)_2_, followed by the nucleation and growth of Ni NPs in the cavities formed by the PPP network. According to the thermal gravimetric analysis (TGA) (Figure S2), the thermal decomposition of Ni(acac)_2_ in the presence of PPP starts at around 190 °C thus indirectly confirming the above scenario.

As it was mentioned above, the synthesis of Ni NPs@PPP by the injection approach was not accompanied by the formation of the cross-linked polymer structure. We assume that at the temperature of the Ni precursor injection (>200 °C), the terminal ethynyl groups (which are necessary for the cyclotrimerization and crosslinking) in the host PPP have already reacted. To verify this assumption, we recorded the FTIR spectrum of the pure polymer after subjecting it to heating at 200 °C for 1 h. The FTIR spectrum showed no bands at 2100 cm^−1^ and 3300 cm^−1^ corresponding to -C≡CH ethynyl end groups (Figure 4, blue line). We propose that the disappearance of ethynyl groups occurs as result of their Diels–Alder cyclocondensation with residual cyclopentadienone fragments of the polymer during heating (for details see the synthetic procedure for PPP in [39]). It is worth noting that the polymer remained soluble through the heating procedure and no gel was formed.

According to the TEM data, the size of the NPs formed by the injection approach exceeds the size of the NPs synthesized by the heat-up method, revealing the key role of the polymer architecture in the NP formation (Figure 2 and Figure 3). For the heat-up approach, the NP growth is mainly restricted by the size of cavities of the polymer network formed during the NP synthesis, while for the “injection” method, only a spatially constrained architecture of the soluble hyperbranched polymer determines the NP size at similar loadings and temperatures. Both types of polymer structure effectively provide supporting and stabilizing functions.

### 2.2. Oxidation State of Ni NPs

**Figure 5:**
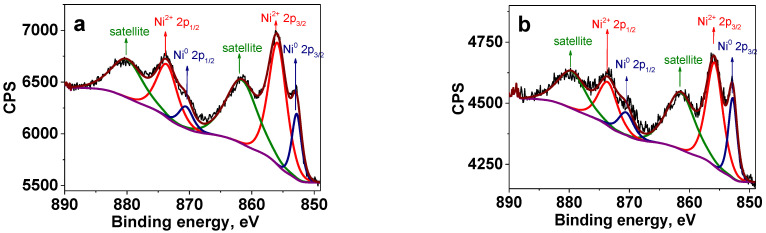
HR XPS of the Ni 2p region for Ni NPs@PPP prepared at 220 °C (**a**) and 280 °C (**b**) by the heat-up method. Deep blue is for Ni^0^, red is for Ni^2+^, green line represents Ni^2+^ satellites, brown is for generated fit, purple is for background and black is for raw data. See Table S1 and Figure S3 for the deconvolution parameters and survey spectrum, respectively.

X-ray photoelectron spectroscopy (XPS) has been utilized for analysis of the surface structure of Ni NPs prepared by the above two methods. The survey spectra of the composites synthesized by the different approaches are presented in Figures S3 and S4. They show the presence of C, O, Ni, and N. Figure 5 displays high resolution (HR) XPS spectra of the samples prepared at 220 °C and 280 °C by the heat-up method. (For NPs prepared at 200 °C, see Figure S5). The binding energies of the Ni 2p_3/2_ major peaks for all three samples are in good agreement with Ni^0^ (852.8 eV) and Ni^2+^ in form of Ni(OH)_2_ (855.9 eV). The Ni 2p_1/2_ peaks at 869.9 eV and 873.7 eV also correspond to Ni^0^ and Ni(OH)_2_, respectively [44,45]. It is worth noting that with an increase in the reaction temperature, the higher fraction of Ni^0^ is formed on the Ni NP surface, indicating partial reduction in Ni^2+^ by products of the acetylacetonate decomposition [46,47]. According to XPS, the ratio of Ni^0^: Ni^2+^ was 1:3.5 for 220 °C and 1:2.4 for 280 °C (see Table S1 for deconvolution parameters).

**Figure 6:**
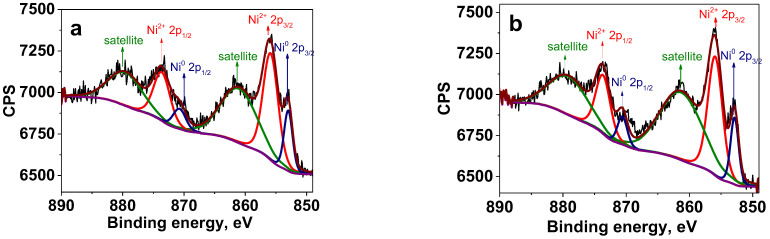
HR XPS of the Ni 2p region of Ni NPs@PPP prepared at 240 °C (**a**) and 280 °C (**b**) by the “injection” method. Deep blue is for Ni^0^, red is for Ni^2+^, green line represents Ni^2+^ satellites, brown is for generated fit, purple is for background, and black is for raw data. See Table S2 and Figure S4 for the deconvolution parameters, and survey spectrum, respectively.

The XPS spectrum of the sample prepared at 220 °C by the injection method (Figure S6) shows only the Ni 2p_3/2_ peak at 855.9 eV assigned to Ni^2+^ [45], indicating no Ni^0^ formation at this temperature. However, as the reaction temperature was increased to 240 °C, 260 °C and 280 °C, the XPS spectra show the Ni 2p_3/2_ peaks at 852.8 eV assigned to Ni^0^, which coexisted with the Ni 2p_3/2_ peaks at 855.9, characteristic for Ni^2+^ in the form of Ni(OH)_2_ [45] (Figure 6). Here, similar to the heat-up approach, the fraction of Ni^0^ on the Ni NP surface increased with the increase in the reaction temperature. For example, at 240 °C the ratio Ni^0^:Ni^2+^ was 1:3.1, while at 280 °C it was 1:2.8 (see deconvolution parameters in Table S2).

**Figure 7:**
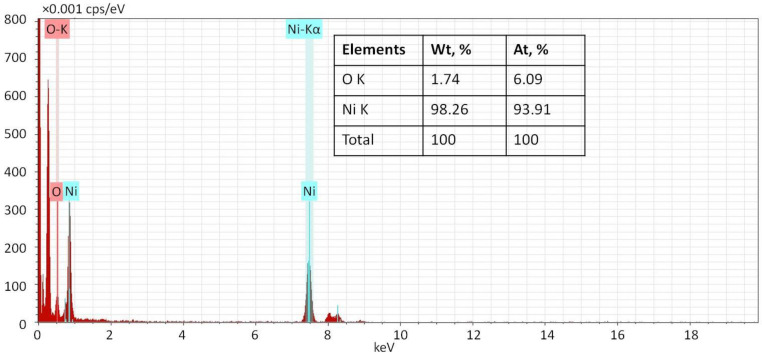
EDX spectrum of Ni NPs@PPP prepared at 280 °C by the heat-up approach.

Considering that XPS is a surface method, energy dispersive X-ray (EDX) analysis was performed additionally to determine the bulk composition of Ni NPs. It showed the predominance of Ni as the Ni:O atomic ratio is 93.91:6.09 (Figure 7). This allows us to suggest that nickel exists primarily in the Ni^0^ state rather than in the oxidized state. The data obtained for the samples prepared at different temperatures are similar (not shown).

**Figure 8:**
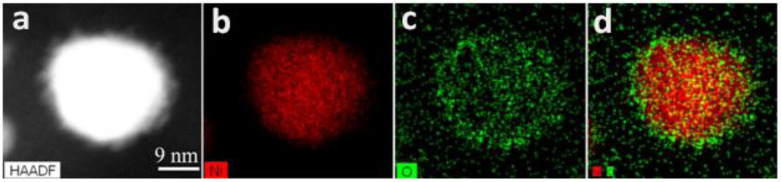
STEM dark-field image (**a**) and EDS maps of Ni (**b**), O (**c**), and Ni-O superposition (**d**) of a single Ni NP@PPP obtained at 280 °C by the heat-up procedure.

To evaluate the position of Ni and O in the NPs, scanning (TEM) (STEM) EDS mapping was performed (Figure 8). The Ni and O maps (Figure 8b,c) repeat the shape of the NP presented in the STEM dark-field image (Figure 8a), indicating that O species are located on/in Ni NPs. The superposition of Ni and O maps (Figure 8d) shows that the oxygen map is slightly larger than the Ni map, revealing a core-shell structure with a higher fraction of oxidized Ni species on the NP surface.

Thus, both approaches led to the formation of predominantly metallic Ni NPs (Ni^0^); however, the heat-up procedure allows NP formation already at 200 °C, while the injection technique requires at least 240 °C for NPs to form. For both procedures, the surface of Ni NPs contains oxidized Ni.

### 2.3. XRD Analysis

**Figure 9:**
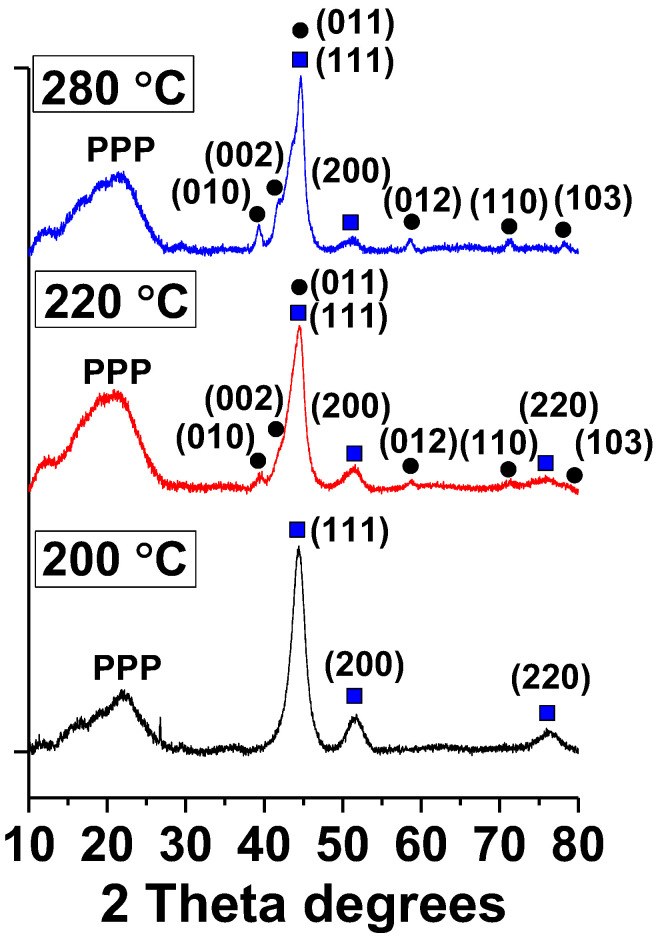
XRD patterns of Ni NPs prepared at 200 °C, 220 °C, 280 °C by the “heat-up” approach. Squares are for the *fcc* phase, while circles indicate the *hcp* phase of Ni^0^. A broad peak at 2*θ* ≈ 20 degrees corresponds to amorphous PPP.

The XRD patterns of Ni NPs prepared by the heat-up approach (Figure 9) at 200 °C demonstrate Bragg peaks at 2θ = 44.55, 51.61, and 76.33, which indicates a cubic Ni phase with the fcc crystalline structure. Ni NPs, which are formed at higher temperatures (220 °C and 280 °C), contain predominantly the Ni^0^ hcp phase, indicating the transformation of the fcc structure to the hcp structure. However, some reflections typical for the fcc structure are also presented (squares at Figure 9). No peaks attributed to Ni(OH)_2_ were observed, indicating its amorphous nature. Ni NPs@PPP prepared by the “injection” method at 240 °C and 280 °C contain mostly the hcp crystalline phase of nickel (Figure S7).

### 2.4. Magnetic Properties of Ni NPs@PPP

**Figure 10:**
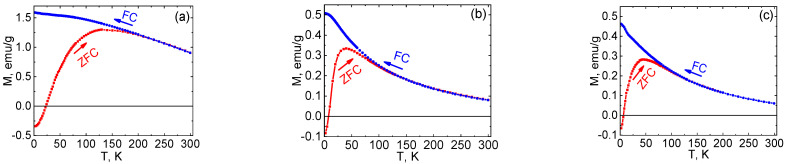
Zero-field-cooled (ZFC) (red line) and field-cooled (FC) (blue line) magnetization curves of Ni NPs@PPP under the applied magnetic field of 100 Oe: H200 (**a**), H280 (**b**), and I280 (**c**).

**Figure 11:**
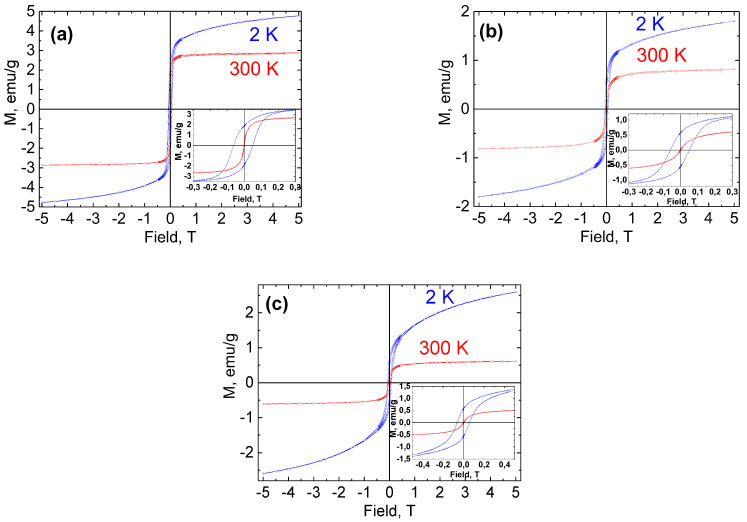
Magnetization versus applied field hysteresis loops measured for Ni NPs@PPP: H_200_ (**a**), H_280_ (**b**), and I_280_ (**c**). The insets show the low field details of the cycles.

Magnetic measurements of Ni NPs@PPP prepared by different methods and at different temperatures were performed to elucidate the effect of these factors on the magnetic behavior of Ni NPs. The temperature-dependent magnetization curves and hysteresis loops are shown in Figure 10 and Figure 11, respectively. The magnetic properties are collected in Table S3. The samples prepared by the heat-up method at 200 °C and 280 °C and by the injection method at 280 °C were designated as H_200_, H_280_, and I_280_, respectively.

ZFC and FC magnetization measurements revealed a distinct magnetic cooling effect for the samples obtained at different temperatures (Figure 10). While the samples prepared at 280 °C by both methods demonstrate a quite similar magnetic behavior (Figure 10b,c), Ni NPs@PPP obtained at 200 °C (Figure 10a) shows the higher magnetization values per gram at 2 K and 300 K in ZFC and FC experiments. Above the blocking temperature, which is a crossing point of the ZFC and FC curves, the magnetization of H_280_ and I_280_ samples decreases quickly and reaches a value close to zero. This negligible magnetization is in contrast with that of the H_200_ sample, which is at least double. The blocking temperatures are 195 and 110 K for the samples obtained at 200 °C and 280 °C, respectively. It is noteworthy that the samples synthesized at 280 °C by two different techniques possess similar blocking temperatures despite the different particle sizes (17.8 ± 2.3 nm and 12.3 ± 1.5 nm for I_280_ and H_280_, respectively). The blocking temperature is known to increase with the increase in the particle size in accordance with the equation T_B_ = KV/25k_B_, where K is the magnetocrystalline anisotropy constant, k_B_ is the Boltzmann constant, and V is the volume of a single particle. However, the results obtained indicate that Ni NPs@PPP do not have a strong dependence of the blocking temperature on the NP sizes and the difference in magnetic properties cannot be explained in terms of size-dependency. Most probably, the magnetic characteristics of the Ni NPs are associated with their crystalline structure. As was demonstrated by XRD, the samples synthesized at 280 °C contain the *hcp* Ni (see Figure 9 and Figure S7), while Ni NPs@PPP prepared at 200 °C contains the *fcc* phase. The similar difference in magnetic properties of *fcc* and *hcp* nickel was observed in an earlier work [48].

The hysteresis loops measured at 2 and 300 K are depicted in Figure 11. All the samples demonstrated hysteretic behavior at 2 K. The dependences M(H) at 2 K do not reach saturation even at a 5 T field, which indicates the presence of noninteracting nickel ions and/or extremely small particles. As it can be seen from Figure 11 and Table S3, the measured coercivity (H_c_) values indicate that Ni NPs@PPP have ferromagnetic properties and exchange bias phenomena at 2 K [49,50]. The exchange shift of the hysteresis loop is most likely observed due to the presence of an oxide (antiferromagnetic) species on the Ni NP surface, which leads to the exchange interaction at the AFM—FM interface. However, the coercivity values measured at 300 K were negligible (in comparison with the low accuracy of setting weak magnetic fields in the superconducting magnet used during measurements) and no hysteresis behavior was observed, which revealed that Ni NPs@PPP are superparamagnetic or soft magnetic at room temperature. Again, the *fcc* and *hcp* Ni NPs demonstrated a difference in magnetic behavior, which become clearer at 300 K. The saturation magnetization (M_s_) values (0.61 and 0.82 emu/g) of the *hcp* Ni NPs were much lower than that of *fcc* (2.87 emu/g). Our results support previous findings [48] that showed the stronger magnetic properties of *fcc* Ni NPs over *hcp* NPs. Nevertheless, all the samples are sensitive to the applied magnetic field and could be easily separated from the reaction solution with an external rare earth magnet at room temperature.

### 2.5. Catalyst Testing in Suzuki-Miyaura Cross-Coupling Reaction

C-C-cross coupling reactions are one of the most widely used methods for the synthesis of valued organic compounds, including pharmaceuticals and bioactive compounds [51]. Ni NPs@PPP prepared by both approaches were tested as catalysts in the model reaction of 4-Br-benzaldehyde (4-BrBA) and phenylboronic acid (PBA) under mild conditions at low Ni NPs loadings (Table 1). The goal of the catalytic study was to assess what approach to preparation of Ni NPs is more appropriate, to provide the best catalytic performance in the above reaction. In addition, the influence of the polymer architecture on the behavior of catalytic NPs in the catalytic reaction, especially, on the stability of the catalytic performance in repeated cycles was evaluated. The samples tested included H_220_ and H_280_ prepared by the heat-up technique at 220 °C and 280 °C, respectively, and I_240_ and I_280_ prepared by the injection method at 240 °C and 280 °C, respectively. All reactions were conducted under similar conditions (see the Experimental section) and the results are collected in Table 1. The catalysts were magnetically recovered after reaction and tested in the six subsequent reaction cycles.

**Figure 12:**
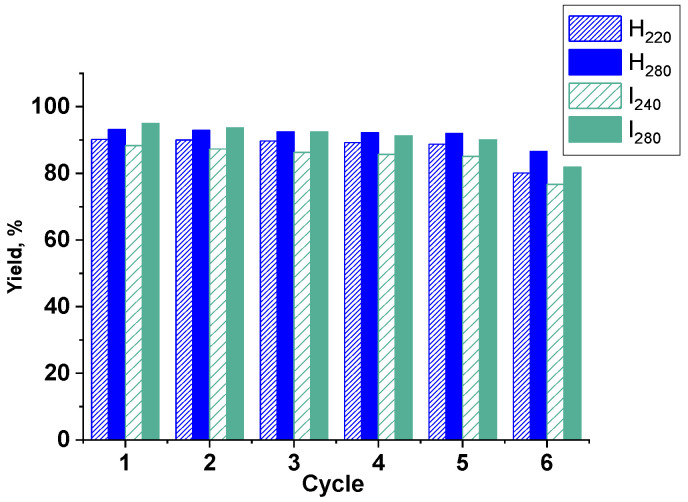
The results of recycling catalytic experiments for Ni NPs@PPP prepared by the heat-up approach at 220 and 280 °C (H_220_ and H_280_) and by the injection method at 240 and 280 °C (I_240_ and I_280_).

The analysis of catalytic data demonstrated that for Ni NPs prepared by both approaches, the yield of the target product is higher when NPs are smaller and contain a higher fraction of Ni^0^ (according to XPS). However, for samples prepared at the lower temperature, the selectivity of the reaction is higher, presumably due to reduced activity of Ni NPs, which is consistent with the low Ni^0^ content on the NPs’ surface (H_220_ and I_240_). With the increase in the amount of Ni^0^ (see H_280_ and I_280_), rates of both homocoupling and cross-coupling reactions increase, which reduces the selectivity. Yet, in case of I_280_, we did not observe a significant drop in selectivity compared with I_240_. We attribute this phenomenon to similar amounts of Ni^0^ in both catalytic NPs (see XPS data) as well as to mostly colloidal nature (NPs stabilized by a soluble polymer) of the nanocomposite prepared by the “injection” procedure. The data presented in Table 1 demonstrate that both approaches to the preparation of Ni NPs@PPP resulted in catalysts with similar catalytic activity (~93–95% yield) in the model Suzuki–Miyaura cross-coupling reaction. These results encouraged us to decrease the Ni loading in the catalytic reaction down to 0.05 mol% for the most active catalysts, H_280_ and I_280_. (The results are marked with asterisk in Table 1). This led to the slower reaction, resulting in 91% and 94% yields of the target coupling product for H_280_ and I_280_, respectively, after 24 h of the reaction, revealing excellent catalytic activity. The stability and reusability of these catalysts in the Suzuki–Miyaura reaction were also remarkable, although not identical and dependent on the catalyst preparation method (Figure 12).

**Figure 13:**
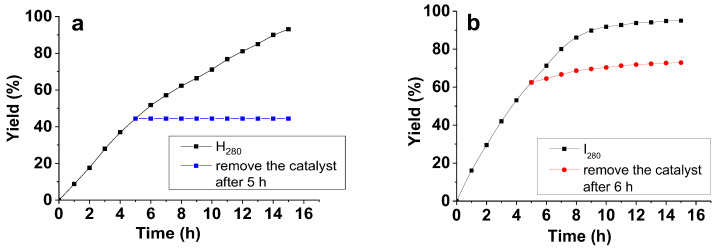
Heterogeneous test for Ni NPs@PPP prepared at 280 °C by the “heat-up” approach (H_280_, **a**) and prepared at 280 °C by the “injection” approach (I_280_, **b**).

While the activity of each catalyst was high enough during five catalytic cycles, the yield of the target product in the case of I_280_ dropped more noticeably than in the case of H_280_ (Table 1 and Figure 12). This drop was even more pronounced during the sixth catalytic cycle. We attribute this effect to the more open and loose architecture of the non-crosslinked hyperbranched PPP support (I_280_), which facilitates Ni leaching from the composite compared to the crosslinked polymer (H_280_). To assess this hypothesis, we performed the heterogeneous test. For this, the reaction of 4-BrBA with PBA was stopped after five hours, followed by the magnetic separation of the catalyst. The reaction mixture without the catalyst was further heated in another flask for additional five hours and analyzed by GC (Figure 13). The results showed a slight increase in the biphenylcarbaldehyde yield for the I_280_ sample, verifying leaching of Ni species. For H_280_, the yield of the reaction product did not change. Thus, the results of the heterogeneous test confirmed a critical effect of the architecture of the catalytic support on the catalyst stability.

These results show promise for Ni NPs@PPP, as they operate in mild reaction conditions and at low catalyst loading, are easily magnetically separable, and preserve high catalytic activity in five consecutive cycles.

## 3. Materials and Methods

### 3.1. Materials

Nickel (II) acetylacetonate (Acros Organics, 97%), benzyl ether (Sigma-Aldrich, 98%), potassium phosphate (Merck, 99%), phenylboronic acid (Merck, 95%), and 4-bromobenzaldehyde (Merck, 99%) were used as received. *N,N*-dimethylformamide (Acros Organics, 99.8%, extra dry) was used without further purification. Acetone (99.5%) and ethanol (99.8%) were purchased from Sigma-Aldrich and used without purification. Hyperbranched pyridylphenylene polymer (PPP) was synthesized as described in [39]. According to size-exclusion chromatography (SEC), the average molecular weight of the polymer was 23,403 g/mol.

### 3.2. Synthesis of Ni NPs

The synthesis of Ni NPs in the presence of the polymer was performed according to the following procedures. For the heat-up approach, 0.257 g (1 mmol) of Ni(acac)_2_, 0.148 g (0.0064 mmol) of pyridylphenylene polymer, and 7 mL of benzyl ether were placed in a round-bottom flask equipped with a magnetic stir bar and a reflux condenser. The molar ratio of PPP to Ni(acac)_2_ was adjusted in several experiments. The mixture was stirred under a flow of high purity argon at room temperature for 15 min. The temperature was then raised to 80 °C to allow the solubilization of reactants and maintained at that temperature for 90 min. The mixture was further heated with a heating rate of 10 °C/min to the desired temperature (200, 220, or 280 °C) and kept there with vigorous stirring for 30 min. After cooling to room temperature, the reaction solution was precipitated into ethanol and washed several times with ethanol and acetone until a colorless supernatant was obtained. The precipitate was separated with a magnet and dried under vacuum.

For injection method, 0.148 g (0.0064 mmol) of PPP was dissolved in 6 mL of benzyl ether in a round-bottom flask equipped with a magnetic stir bar, reflux condenser, and septa. The mixture was stirred under a flow of argon at 80 °C for 90 min. 0.1285 g (0.5 mmol) of Ni(acac)_2_ was dissolved in 1 mL of benzyl ether in a separate flask under an inert atmosphere at 90 °C. The polymer solution was heated at 10 °C/min to the desired temperature (200 °C, 220 °C, 240 °C, 260 °C, or 280 °C). Upon reaching the temperature, a Ni(acac)_2_ solution was quickly injected into hot polymer solution and the reaction mixture was further stirred at that temperature for 30 min. After cooling to room temperature, the reaction solution was precipitated into ethanol and washed several times with ethanol and acetone until the colorless supernatant was obtained. The precipitate was separated with magnet and dried under vacuum.

### 3.3. General Procedure for Suzuki-Miyaura Cross-Coupling Reaction

Typically, 4-bromobenzaldehyde (0.0925 g, 0.5 mmol), phenylboronic acid (0.091 g, 0.75 mmol), potassium phosphate (0.3185 g, 1.5 mmol), Ni NPs@PPP composite (0.5 mol% or 0.05 mol% of Ni) and DMF (1.5 mL) were placed in a Schlenk flask and well dispersed using ultrasonic bath prior the reaction. The reaction was performed under argon atmosphere upon vigorous stirring (550 rpm) at 100 °C. The flask was equipped with a condenser. The reaction was monitored by gas chromatography (GC) using Chromatec-Crystal 5000.2 chromatograph equipped with a flame ionization detector (FID) and a DB-1 column (length = 100 m, inner diameter 0.25 mm and film thickness = 0.5 µm). Nitrogen was used as a carrier gas. The temperature program for the GC analysis was raised from 200 to 270 °C at 10 °/min and kept at 270 °C for 20 min. The detector temperature was 290 °C and the injector temperature was 295 °C. Products were identified by comparison with authentic samples.

### 3.4. Catalyst Recycling Experiments

After the experiments, catalysts were separated from the reaction mixtures using a rare-earth magnet, washed with EtOH (50 mL) and water until a neutral pH was obtained. The catalysts were washed again with EtOH (30 mL) and placed in chloroform (30 mL). The resulted suspensions were sonicated for 15 min, the chloroform was removed and the solid was dried at 65 °C to constant weight. The catalysts obtained were used in repetitive Suzuki–Miyaura reactions.

### 3.5. Characterization

Transmission electron microscopy (TEM), scanning transmission electron microscopy (STEM), electron diffraction (ED), and energy-dispersive X-ray (EDX) microanalysis were carried out in an Osiris TEM/STEM (Thermo Fisher Scientific, Waltham, MA, USA) equipped with a high-angle annular dark field detector (HAADF) (Fischione, Export, PA, USA) and an X-ray energy dispersive spectrometer Super X (ChemiSTEM, Bruker, Billerica, MA, USA) at an accelerating voltage of 200 kV. Specimens for TEM, STEM, and EDXS studies were prepared by placing a drop of the sample suspension on a Cu grid coated with a Lacey carbon film. The particle size distribution histograms were obtained for approximately100 particles with Scion Image software.

Powder X-ray diffraction patterns were recorded using a Rigaku MiniFlex600 diffractometer (Rigaku Corporation, Tokyo, Japan) with Si Ka radiation (40 kV, 15 mA, Ni-Kß filter) in the angular range 2θ = 20–80°. A scanning step was set to be 0.02° and a speed was 0.5 °/min. Identification was performed with the PDXL software (Rigaku Corporation, Tokyo, Japan) using the ICDD PDF-2 database (2017).

X-ray photoelectron spectroscopy (XPS) data were obtained using Axis Ultra DLD (Kratos) spectrometer with a monochromatic Al Kα radiation. All the data were acquired at X-ray power of 150 W. Survey spectra were recorded at an energy step of 1 eV with an analyzer pass energy 160 eV, and high-resolution spectra were recorded at an energy step of 0.1 eV with an analyzer pass energy 40 eV. Samples were out gassed for 180 min before analysis. The data analysis was performed by CasaXPS.

Ni content of the composites were obtained from X-ray fluorescence (XRF) measurements using a Zeiss Jena VRA-30 spectrometer equipped with a Mo anode, a LiF200 crystal analyzer, and a SD detector.

The magnetic susceptibility measurements were obtained with the use of a Quantum Design PPMS-9 susceptometer. This experimental system works between 1.8 and 400 K for DC-applied fields ranging from −9 to 9 T. Measurements were performed on analytically pure polycrystalline samples introduced in polyethylene bags. The magnetic data were corrected for the sample holder and the bag.

Thermal gravimetric analysis (TGA) was performed on Shimadzu DTG-60H at a heating rate of 10 °/min under an argon atmosphere.

FTIR spectra were recorded on a Vertex 70 V Fourier spectrometer (Bruker, Germany) using a Pike ATR accessory with a diamond crystal (Nicolet, USA); the ATR spectra were averaged from 128 scans over a range of 400–4000 cm^−1^ with a resolution of 4 cm^−1^.

## 4. Conclusions

We developed two different types of Ni NPs@PPP composites using the heat-up and injection procedures for the thermal decomposition of the Ni precursor in the presence of PPP. While the heat-up procedure provided the formation of Ni NPs in the cross-linked polymer matrix, the injection protocol led to Ni NPs stabilized by the soluble hyperbranched polymer. A thorough characterization of the nanocomposites developed showed that, for both approaches, an increase in the decomposition temperature leads to an increase in the Ni^0^ fraction in Ni NPs, while the Ni NP size is larger for the injection method, revealing a significant role of the polymer architecture in NP formation. The crystalline structure of Ni NPs mostly depends on the reaction temperature. It was demonstrated that the increase in temperature from 200 °C to 280 °C led to the transformation of the *fcc* to an *hcp* structure. At the same time, Ni NPs prepared by both procedures at 280 °C contains mostly the *hcp* phase. The differences of the crystalline structure of Ni NPs are reflected in the different magnetic behavior of nanocomposites, but all nanocomposites are magnetically recoverable.

Ni NPs@PPP demonstrated an excellent catalytic activity (93–95% yield) in the model Suzuki–Miyaura reaction at low catalytic loading and in mild conditions, allowing for catalyst magnetic recovery and multiple reuse (in five catalytic cycles) without a noticeable loss of activity. Thus, a hyperbranched polymer may serve as a universal platform for manufacturing effective catalytic NPs. Such NPs can be either colloidal species stabilized by a soluble hyperbranched macromolecule or locked in a spatially linked polymer network for heterogeneous catalysis, depending on the preparation procedure employed.

## Figures and Tables

**Table 1:** Results of the catalytic performance of Ni NPs@PPP in the Suzuki–Miyaura reaction ^a^.

		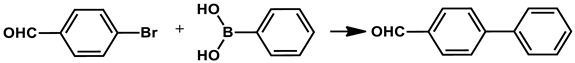					
**#**	**Sample Name**	**Time of Reaction, h**	**Conversion, %**	**Selectivity, %**	**Yield ^b^, %**	**Yield ^c^, % after 5th Cycle**	**Yield ^c^, % after 6th Cycle**
1	H_220_	6	53	78	41.3	88.8	80.1
2		15	93	97	90.2		
3	H_280_	6	69	75	51.8	92.0	86.6
4		15	99	94	93.1		
5	I_240_	6	71	83	58.9	85.1	76.7
6		15	92	96	88.3		
7	I_280_	6	81	88	71.3	90.1	81.9
8		15	100	95	95.0		
9 *	H_280_	15	54.5	81.3	44.3	87.1	82.7
		24	97.3	93.7	91.2		
10 *	I_280_	15	65.2	85.3	55.6	86.3	77.1
		24	98.0	96.0	94.1		

^a^ Reaction conditions: 4-BrBA (0.5 mmol), PBA (0.75 mmol), catalyst (0.5 mol%), K_3_PO_4_ (1.5 mmol), DMF 1.5 mL, 100 °C; ^b^ Yield was calculated as product of conversion and selectivity obtained by gas chromatography. ^c^ Yield calculated after 15 h. * The experiments were conducted with the reduced catalyst amount (0.05 mol%).

## Data Availability

The data presented in this study are available upon request from the corresponding author.

## Supplementary Materials

The following supporting information can be downloaded at: https://www.mdpi.com/article/10.3390/ijms232213874/s1.
