# Supplementary material for: Ni Nanoparticles Stabilized by Hyperbranched Polymer: Does the Architecture of the Polymer Affect the Nanoparticle Characteristics and Their Performance in Catalysis?
Source: Int J Mol Sci. 2022 Nov 10;23(22):13874. doi: 10.3390/ijms232213874 (PMC9696000; doi:10.3390/ijms232213874)
Supplement: Supplementary file 1 [file ijms-23-13874-s001.zip › ijms-1966391-supplementary-done.pdf]

International Journal of
Molecular Sciences

MDPI

Supplementary information

Ni Nanoparticles Stabilized by Hyperbranched Polymer: Does Architecture of the Polymer Affect The Nanoparticle Characteristics and Their Performance in Catalysis?

Svetlana A. Sorokina, Nina V. Kuchkina, Mariam G. Ezernitskaya, Alexey V. Bykov, Alexander L. Vasiliev, Nikolay N. Efimov and Zinaida B. Shifrina

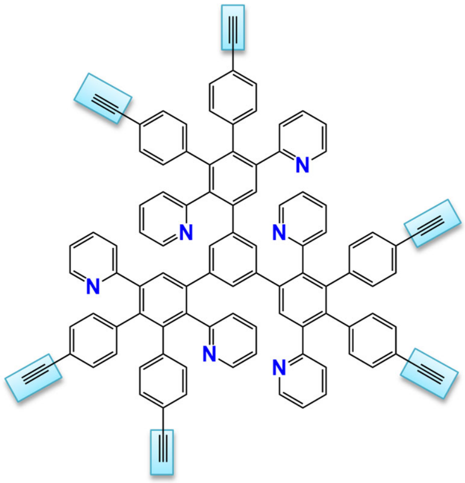

Figure S1. Pyridylphenylene dendrimer of the first generation used as a model compound for the PPP structural unit in the FTIR experiments.

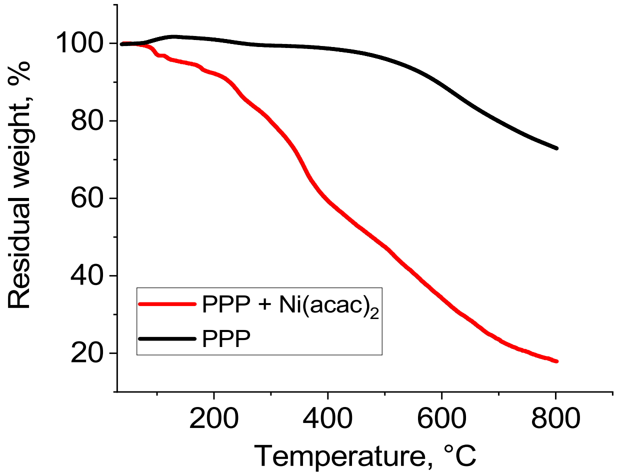

Figure S2. TGA profiles of PPP (black line) and its complex with nickel acetylacetonate (red line) under argon atmosphere.

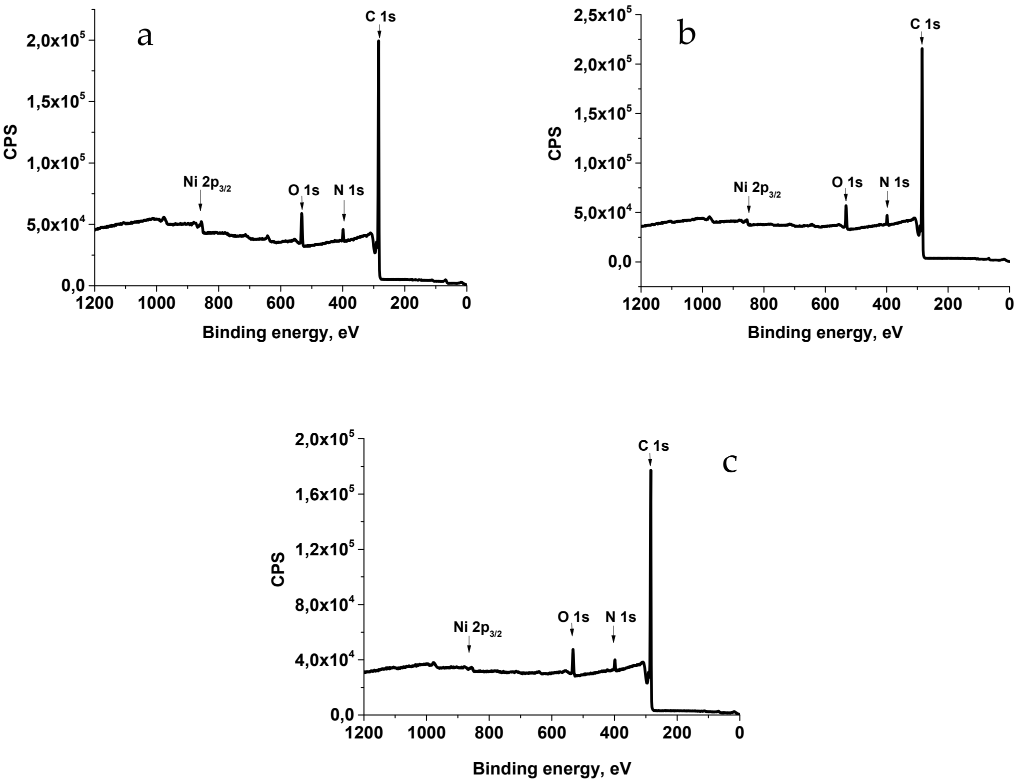

Figure S3. XPS survey spectra of Ni NPs@PPP obtained at 200°C (a), 220°C (b) and 280 °C (c) by the heat-up method.

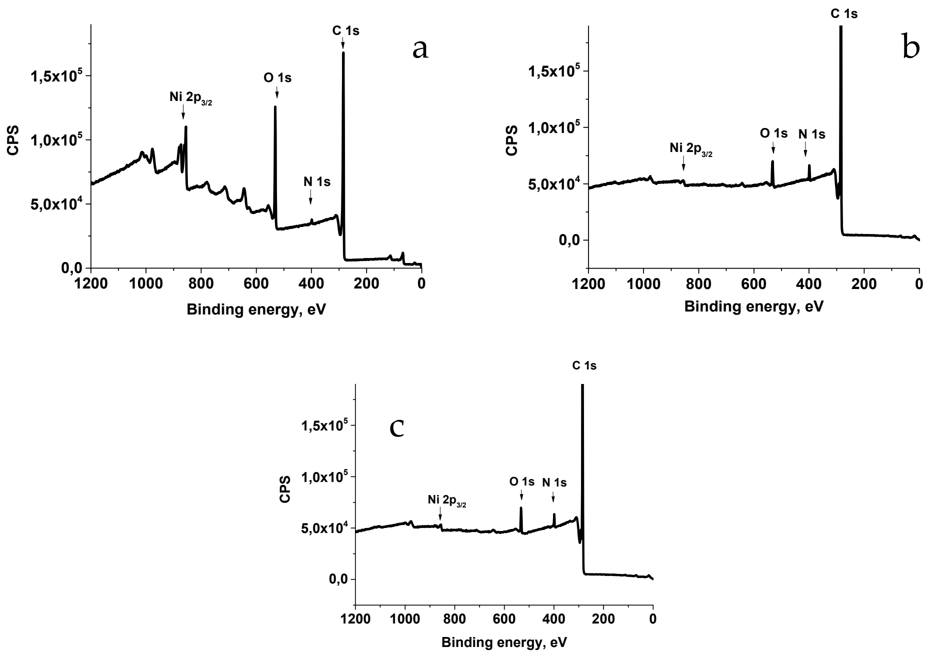

Figure S4. XPS survey spectra of Ni NPs@PPP obtained at 200°C (a), 220°C (b) and 280°C (c) by the injection method.

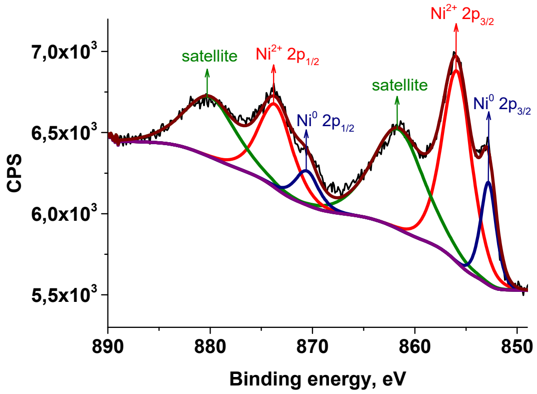

Figure S5. HR XPS of the Ni 2p region for Ni NPs@PPP obtained at 200 °C by the heat-up approach. Deep blue is for Ni^{0}, red is for Ni $^{2+}$, green line represents Ni^{2+} satellites, brown is for generated fit, purple is for background and black is for raw data.

Table S1. Fitting parameters for the HR XPS Ni 2p spectrum of Ni NPs@PPP obtained at different temperatures by the heat-up method.

Sample name/ temperature of the synthesis	Band	Position, eV	FWHM, eV	%Area
H_{200}/ 200 °C	Ni $2p_{3/2}$	855.84	2.74	25.46
	Ni 2p sat	861.05	6.95	33.25
	Ni $2p_{1/2}$	873.58	3.2	12.60
	Ni 2p sat	879.30	6.85	18.73
	Ni $2p_{3/2}$	852.94	1.74	6.55
	Ni $2p_{1/2}$	870.68	2.27	3.41
H_{220}/ 220 °C	Ni $2p_{3/2}$	855.58	3.41	29.48
	Ni 2p sat	861.59	6.00	27.05
	Ni $2p_{1/2}$	873.62	3.91	14.74
	Ni 2p sat	880.00	6.00	16.23
	Ni $2p_{3/2}$	852.80	1.80	8.33
	Ni $2p_{1/2}$	870.54	2.70	4.17
H_{280}- 280 °C	Ni $2p_{3/2}$	855.82	3.20	27.15
	Ni 2p sat	861.43	5.50	25.73
	Ni $2p_{1/2}$	873.56	3.81	13.57
	Ni 2p sat	879.60	5.50	16.74
	Ni $2p_{3/2}$	852.80	1.70	11.20
	Ni $2p_{1/2}$	870.54	3.00	5.60

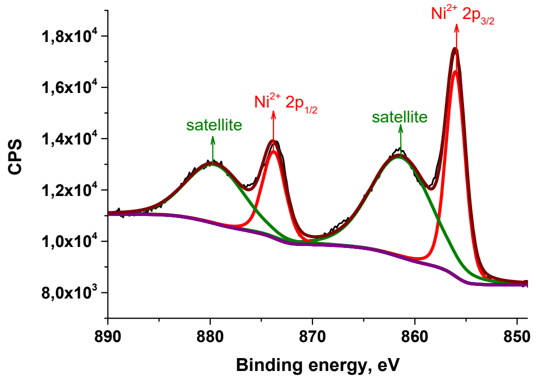

Figure S6. HR XPS of the Ni 2p region for Ni NPs@PPP obtained at 220 °C by the injection approach. Red line is for Ni $^{2+}$, green line represents Ni^{2+} satellites, brown is for generated fit, purple is for background and black is for raw data.

Table S2. Fitting parameters for the HR XPS Ni2p spectrum of Ni NPs@PPP obtained at different temperatures by the injection method.

Sample name/ temperature of the synthesis	Band	Position, eV	FWHM, eV	%Area
I_{220}/ 220 °C	Ni $2p_{3/2}$	856.01	2.25	25.73
	Ni 2p sat	861.33	7.03	39.34
	Ni $2p_{1/2}$	873.75	2.66	12.87
	Ni 2p sat	879.51	6.68	22.07
I_{240}/ 240 °C	Ni $2p_{3/2}$	855.87	2.89	25.62
	Ni 2p sat	861.02	6.94	31.33
	Ni $2p_{1/2}$	873.61	3.18	12.81
	Ni 2p sat	879.54	6.43	17.83
	Ni $2p_{3/2}$	852.98	1.65	8.28
	Ni $2p_{1/2}$	870.72	2.47	4.14
I_{280}/ 280 °C	Ni $2p_{3/2}$	855.93	2.71	21.52
	Ni 2p sat	861.30	7.96	37.15
	Ni $2p_{1/2}$	873.67	2.74	11.09
	Ni 2p sat	879.10	7.20	19.09
	Ni $2p_{3/2}$	852.86	1.52	7.65
	Ni $2p_{1/2}$	870.60	1.82	3.49

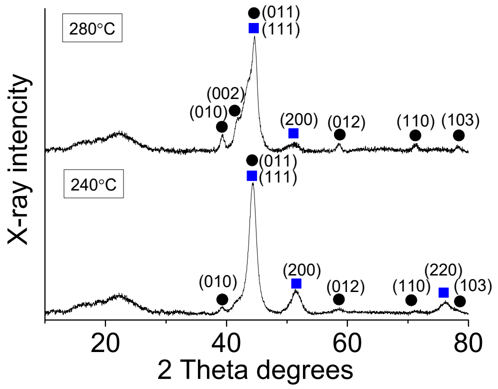

Figure S7. XRD patterns of Ni NPs@PPP prepared at 240 and 280 °C by the injection approach. Circles indicate the *hcp* phase of Ni, while squares are for the *fcc* phase.

Table S3. Magnetic properties of Ni NPs@PPP prepared by the heat-up method at 200 ˚C and 280 ˚C (H_{200} and H_{280}), and by the injection method at 280 ˚C (I_{280}).

**Sample**	**Hysteresis parameters**			**M_S(5T)**		**M(2K, 100Oe)**		**M(300K, 100Oe)**	**T_{max}**	**T_B**
**Units**		**Oe**				**emu/g**				**K**
	2 K	H_C	shift	2 K	300 K	ZFC	FC		ZFC	
**H_{200}** *fcc* Ni, 15.2±2.0 nm	-619 +507	563	-56	4.78	2.87	-0.34	1.59	0.91	130	195
**H_{280}** *hcp* Ni, 12.3±1.5 nm	-580 +514	547	-33	1.80	0.82	-0.08	0.51	0.08	40	110
**I_{280}** *hcp* Ni, 17.8±2.3 nm	-620 +590	605	-15	2.59	0.61	-0.06	0.46	0.06	46	110
